# SegX-Net: A novel image segmentation approach for contrail detection using deep learning

**DOI:** 10.1371/journal.pone.0298160

**Published:** 2024-03-05

**Authors:** S. M. Nuruzzaman Nobel, Md. Ashraful Hossain, Md. Mohsin Kabir, M. F. Mridha, Sultan Alfarhood, Mejdl Safran

**Affiliations:** 1 Department of Computer Science and Engineering, Bangladesh University of Business and Technology, Dhaka Bangladesh; 2 Superior Polytechnic School, University of Girona, Girona, Spain; 3 Department of Computer Science, American International University-Bangladesh, Dhaka, Bangladesh; 4 Department of Computer Science, College of Computer and Information Sciences, King Saud University, Riyadh, Saudi Arabia; Prince Mohammad Bin Fahd University, SAUDI ARABIA

## Abstract

Contrails are line-shaped clouds formed in the exhaust of aircraft engines that significantly contribute to global warming. This paper confidently proposes integrating advanced image segmentation techniques to identify and monitor aircraft contrails to address the challenges associated with climate change. We propose the SegX-Net architecture, a highly efficient and lightweight model that combines the DeepLabV3+, upgraded, and ResNet-101 architectures to achieve superior segmentation accuracy. We evaluated the performance of our model on a comprehensive dataset from Google research and rigorously measured its efficacy with metrics such as IoU, F1 score, Sensitivity and Dice Coefficient. Our results demonstrate that our enhancements have significantly improved the efficacy of the SegX-Net model, with an outstanding IoU score of 98.86% and an impressive F1 score of 99.47%. These results unequivocally demonstrate the potential of image segmentation methods to effectively address and mitigate the impact of air conflict on global warming. Using our proposed SegX-Net architecture, stakeholders in the aviation industry can confidently monitor and mitigate the impact of aircraft shrinkage on the environment, significantly contributing to the global fight against climate change.

## 1 Introduction

**Fig 1:**
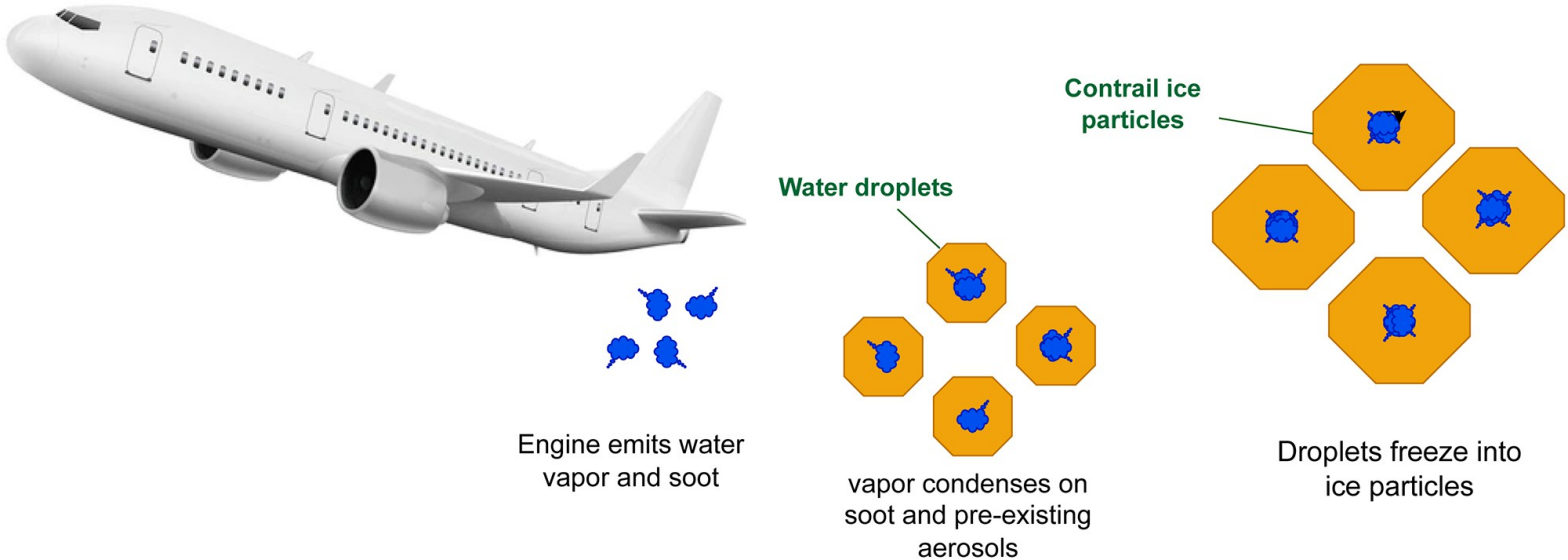
Our research study observed that high-altitude ice cloud formation is facilitated by the condensation of water droplets on aircraft engine soot and other aerosols.

The skies above are dynamic ecosystems that react to different natural and artificial influences rather than just being empty canvases for atmospheric occurrences. Aircraft engines are among the latter and have drawn interest because of how they affect the atmosphere. More than half of all aviation’s climate-related emissions come from contrails, which exacerbate the effects of global warming [1]. High-altitude aircraft engines produce exhaust gases that may condense into contrails, observable trails. In the setting of climate change and environmental study, these long, wispy structures have drawn much attention. Contrails are complicated objects with wide-ranging effects; they are not merely ephemeral traces in the sky. On the one hand, they support the Earth’s atmosphere’s radiative forcing, which has a cooling and warming impact. They may deflect sunlight and retain emitted longwave radiation due to their microphysical characteristics and the ice crystals they contain, which can change the planet’s energy balance. Contrails are crucial in affecting climate dynamics, much like their natural counterparts, normal clouds. Contrails also have conflicting impacts, so how they affect the climate must be clarified. In Fig 1, an illustration showcases key components of high-altitude ice cloud formation: engine-emitted water vapor and soot condensing on pre-existing aerosols, resulting in the presence of frozen droplets and contrail ice particles. Due to the existence of ice crystals, they may simultaneously strengthen the greenhouse effect, which traps heat while reflecting sunlight and having cooling effects. Underscoring the complex connection between contrails and climate, research on how these opposing factors balance out is still underway. Scientists are attempting to determine how and to what degree engine designs, various fuels, and atmospheric conditions contribute to climate change in light of the increased air traffic causing a rise in aircraft emissions of contrails during the last two decades [2]. Despite efforts to curb emissions, the stability of these figures over the last decade emphasizes the pressing need for immediate and effective action [3]. Our research is driven by the urgent need to address the environmental consequences of aircraft contrails and their association with emissions. We recognized the need for an innovative approach that transcends conventional methods and harnesses the power of artificial intelligence to achieve exceptional results. Enter SegX-Net, a segmentation architecture tailored for contrail analysis. Unlike traditional approaches, SegX-Net capitalizes on a unique fusion of deep learning techniques. The basis of our research is a crucial environmental concern: the influence of aircraft contrails on climate change. Due to a substantial increase in air traffic, contrails significantly contribute to emissions and global warming. Conventional approaches must be more comprehensive in dealing with the complexities of identifying contrails from satellite images. Therefore, our primary objective is to propose a groundbreaking solution. We selected to modify DeeplabV3+, incorporating ResNet-101 as the backbone. The choice is based on the exceptional capability of ResNet-101 to extract delicate characteristics, which are crucial for deciphering the complicated patterns of contrails in satellite photos. Along with the acclaimed DeeplabV3+, we thoroughly compare well-known models like U-Net, U-Net++, Attention U-Net, Trans U-Net, Res U-Net, and Uc Trans U-Net. The dataset used in this research paper focuses on aircraft contrails, which are clouds of ice crystals formed in aircraft engine exhaust and contribute to global warming by trapping heat in the atmosphere.

The main contributions of the paper include:

- Introduced the SegX-Net architecture, a modified version of DeepLabV3+ with ResNet-101 integration, by customizing the encoder part of the network and leveraging transfer learning, leading to highly accurate and detailed contrail segmentation.
- The research emphasizes the significance of accurate aircraft contrail detection. By providing an advanced image segmentation solution, SegX-Net contributes to the understanding and mitigating contrail-induced environmental impact.
- Through extensive experimentation and evaluation, the study demonstrated that SegX-Net outperforms traditional DeepLabV3+ models with VGG16, VGG19, VIT, Xception, MobileNetV2, ResNet-18, ResNet34, and ResNet101 backbones. Our proposed architecture got exceptional F1-scores and Intersection over Union (IoU) values, indicating its precision, recall, and segmentation quality.

Our research paper contributes to understanding aircraft contrails’ environmental impact and proposes an enhanced architecture for accurate contrail identification through image segmentation. By integrating SegX-Net, we tried to fill a crucial research gap in accurately identifying and analyzing aircraft contrails.

The remainder of the paper is organized as follows. In Section 2, we explore the body of literature in-depth, looking at several image segmentation techniques and their uses. Section 3 describes the dataset description, preprocessing procedures, SegX-Net design, and contrail formation. Analysis of efficiency, insights into parameters, and comparison experiments about assessment measures are discussed in Section 4. Section 5 gives an in-depth discussion of the interpreted findings, including the implications for contrail detection and image recognition of the climate. Finally, Section 6 summarizes our contributions, highlights the importance of SegX-Net, and suggests possibilities for further study.

## 2 Related works

The foundation of any scientific endeavor lies in building upon the existing body of knowledge. In contrail detection using image segmentation, a thorough exploration of related studies is essential to position our work within the broader landscape. This section presents a comprehensive overview of the relevant literature, from image segmentation techniques to the intricate relationship between contrails and climate impact. By delving into these studies, we gain valuable insights that contribute to the foundation of our novel approach, SegX-Net, designed to transform contrail detection through advanced deep learning techniques. Global warming arises from a mix of natural and human-emitted gases that trap heat, causing the Earth’s temperature to increase. These greenhouse gases, like carbon dioxide and methane, obstruct the average energy radiation balance. In the realm of air transport’s environmental effects, aside from emissions and noise, the impact of contrails on the Earth’s radiation balance is a concerning area that needs more comprehensive understanding and precise data [4]. Previous studies have explored various strategies, such as operational changes in air traffic control, to mitigate contrail-induced greenhouse effects. While some investigations focus on the potential benefits of altering cruise flight levels, others acknowledge the complexity and uncertainty surrounding contrail formation and climate impact, underscoring the ongoing need for comprehensive research in this domain [5]. Identifying contrails in aerial images is a difficult task since they closely resemble natural cirrus clouds and undergo form variations over time [6]. Paoli et al. [7] discovered that in an aircraft regime, at lower temperatures and greater humidity, contrails begin to develop near the engine’s edge. While successful in predicting contrail formation, challenges persist in predicting persistence due to humidity uncertainties. The study delves into predicting contrail formation, persistence, and radiative forcing through aviation weather forecasts. Notably, the paper suggests considering the ambient atmosphere’s dynamics to predict strong contrails. This work contributes vital insights into mitigating aviation’s climate impact and underscores the need for refining contrail prediction methods to combat climate change [8]. An instrumental tool in this endeavor is image segmentation applied to satellite and aerial imagery, enabling the identification of critical climate change contributors like deforestation, urban heat islands, and melting glaciers. Precise segmentation of these regions empowers targeted interventions to mitigate the effects of global warming [9]. U Schumann et al. [10] investigated strategies to mitigate aviation’s climate impact through optimized flight routes considering contrail formation and fuel consumption. It introduces climate-optimized routing using the Contrail Cirrus Simulation Prediction tool (CoCiP) and discusses its potential to reduce global warming effects. The study addresses the radiative forcing of the contrail-induced cirrus cover and highlights the need for further validation and refinement of the CoCiP model for accurate contrail prediction. This research aligns with the broader discourse on aviation’s environmental consequences and emphasizes the significance of route optimization for climate protection. In another case, the paper of [11] comprehensively reviews climate change mitigation strategies, encompassing conventional efforts, negative emissions technologies, and radiative forcing geoengineering. It underscores the insufficiency of conventional mitigation to achieve Paris Agreement targets and explores alternative routes. The study highlights the importance of practical solutions like biogenic-based sequestration techniques, which require policy support, carbon pricing mechanisms, and increased research funding for effective implementation. Also, the study of K Segl et al. [12] introduces a novel approach for detecting small objects in high-resolution satellite imagery by combining supervised shape classification with unsupervised image segmentation iteratively. It emphasizes the significance of shape contrast and object size for accurate detection and discusses potential enhancements through multispectral or hyperspectral imagery. This approach holds practical implications for applications like urban monitoring and vegetation analysis, addressing the challenge of object detection in high-resolution panchromatic satellite images. A pioneering study by JP Hoffman et al. [13] introduces an innovative application of Convolutional Neural Networks (CNNs) in contrail detection within satellite imagery. Repurposing the U-Net architecture, initially developed for detecting sea ice leads, this approach accurately identifies contrails through image segmentation. Furthermore, a cooperative strategy utilizing Fuzzy C-means and Self-Organizing Maps attains high accuracy in segmenting satellite images [14]. Researching global cloud and aerosol properties, radiative energy balance, 3D cloud morphology, and infectious disease risk due to climate fluctuations highlighted the importance of studying contrails’ effects on radiative balance and cloud formation in various contexts. The proposed multistep protocol for contrail detection and segmentation in AVHRR images shows promise in identifying contrail properties yet acknowledges challenges in detecting certain types and improving algorithm precision [15]. The study also revealed that converting the RGB color space to HSV enhanced segmenting satellite images, indicating the practical utility of color space transformations in this context [16]. The paper by Andre L. Barbieri et al. [17]presents an entropy-based image segmentation method for color images from Google Earth, enabling automated monitoring of ecological and geographical changes. It underscores the significance of color information for precise segmentation and highlights applications in disaster mapping, climate change monitoring, and ecological studies. The approach’s potential for improvement via window size adjustments and complementary statistical measures is also emphasized. An automatic algorithm combining watershed segmentation and region merging demonstrates promising performance on Google Earth images [18]. Studies revealed that mathematical morphology and watershed transformation algorithms offer segmentation benefits, yet they grapple with challenges such as over-segmentation and computational complexity [19]. B Dezso et al. [20] presents a comprehensive review of graph-based image segmentation methods applied to satellite image classification. It evaluates four algorithms and discusses their theoretical foundations, implementation details, and potential improvements. The study highlights the significance of image segmentation in remote sensing for land cover identification and suggests avenues for future research to enhance algorithm performance and practical application. Leveraging geostationary satellite imagery, weather data and air traffic information, these studies offer insights into contrail evolution and climate impact. Deep learning techniques like instance segmentation are explored for efficient detection. Integration of multiple observation methods and identification of contrail-producing aircraft contribute to advancing contrail research for climate validation and modeling improvement [21]. Recently, there has been a significant increase in research efforts to improve image segmentation methods by using deep learning techniques. This is due to the impressive performance of deep learning models in visual tasks [22, 23].

Advancements in image processing have led to the emergence of image segmentation techniques, prominently in medical and satellite imagery domains. This paper introduces and evaluates three methods for satellite image segmentation: K-means Clustering, Thresholding Technique, and Active contour. By assessing their performance using parameters like Segmentation Accuracy and Correlation Ratio, the study aids in identifying practical options for satellite image analysis. The proposed Active Contours technique exhibits promising results, highlighting its potential for real-world implementation [24]. In the context of advancing semantic segmentation techniques, this study of [25] introduces an innovative algorithm addressing accuracy and object boundary segmentation challenges. The algorithm demonstrates improved performance in segmenting high-resolution images by leveraging multi-level cascading residual structures and multiple loss function constraints. While the experimental results on Cityscapes and CamVid datasets are promising, comprehensive analysis of its applicability, comparative assessments, and real-world implications remain avenues for future exploration. Recently, McCloskey et al. [26] conducted research and provided a restricted set of human-labeled Landsat photographs for the scientific community. In another recent study by Ng et al. [27], a comprehensive effort was made to create an open dataset for contrails observed over the United States. This was achieved by utilizing satellite footage from the GOES-16 satellite.

The subsequent unveiling of SegX-Net’s architecture, training process, and evaluation metrics in this paper is a significant step towards comprehending and mitigating contrail impacts on global warming, revolutionizing climate research. This paper critically reviews image segmentation methods to identify the most suitable techniques for contrail identification. Thus, we propose using an innovative AI-driven technique called SegX-Net for accurate contrail identification. Through this approach, we aim to revolutionize climate research by providing a powerful tool for monitoring and mitigating the effects of aircraft contrails on our environment.

## 3 Methodology

**Fig 2:**
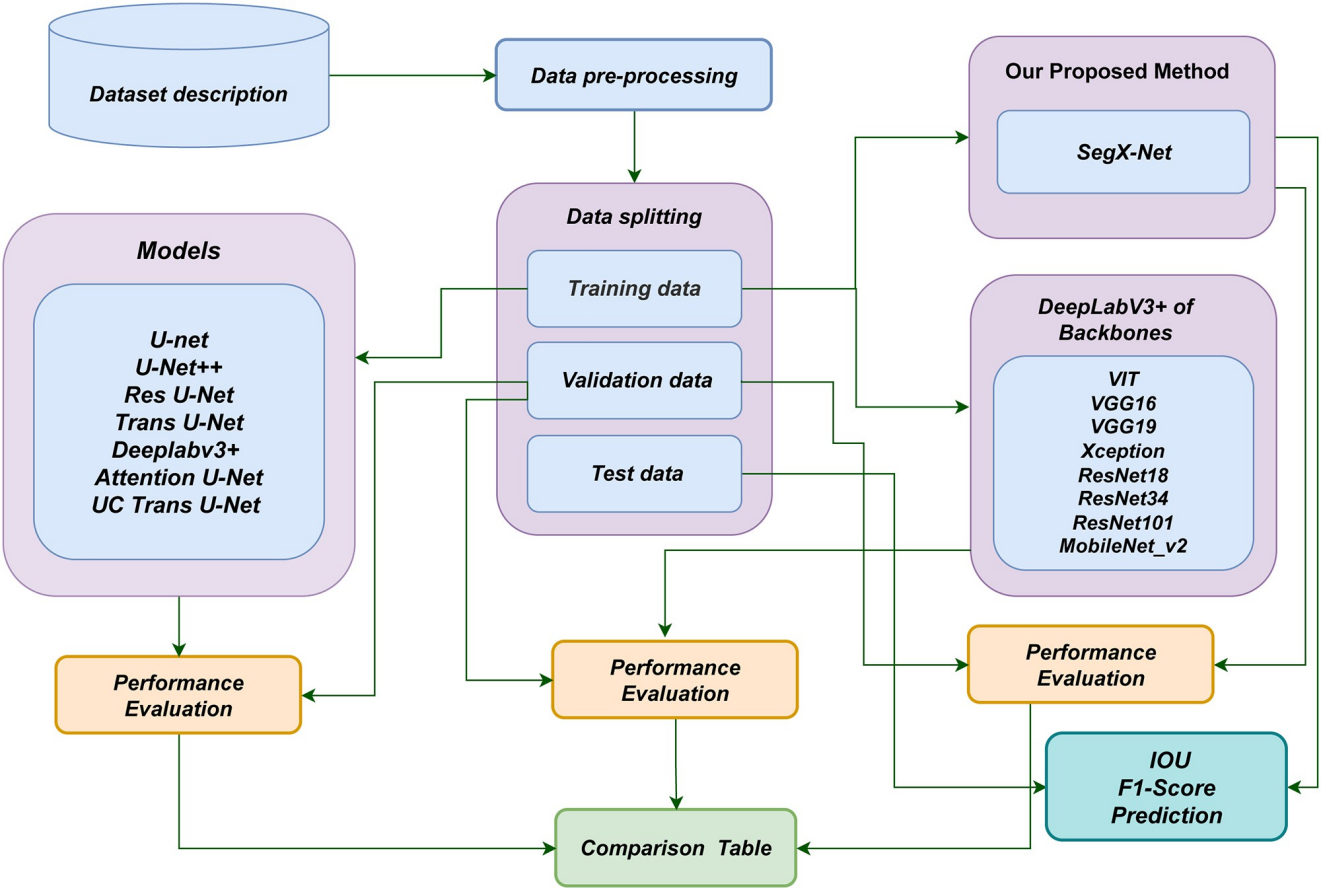
Illustration of the systematic framework employed in this study that visually represents the step-by-step process of the proposed approach.

The research methodology is organized into interconnected phases, illustrated in Fig 2, to enhance contrail detection efficiency through the innovative SegX-Net architecture. It begins with meticulous data preprocessing and splitting into training and validation subsets. The dataset is split into training and valid subsets, enabling unbiased SegX-Net assessment. Crucial backbone network models, including VGG16 [28], VGG19 [29], VIT [30], Xception [31], ResNet18 [32], ResNet34 [33], ResNet101 [34] and MobileNetv2 [35], establish the foundation for SegX-Net.

Simultaneously, the architecture and backbones analyze the training subset, refining image segmentation precision. The subsequent phase involves comprehensive performance evaluation, leading to a succinct comparison table showcasing the contrail detection prowess of each backbone under SegX-Net. This methodical progression harnesses SegX-Net’s power to redefine contrail detection precision, contributing to advancements in this vital field. UNet and DeepLab are top-rated models in the field of aerial photography due to their exceptional performance in image segmentation tasks and their ability to effectively include both global and local information [6]. We used the DeepLabV3+ architecture as our segmentation network during this study. An improvement on the Deeplabv3 architecture, Deeplabv3+ has a more streamlined and effective decoding module for improving semantic segmentation performance and refining feature information [36]. When it comes to segmentation, Deeplabv3+ outperforms the previous Deeplab-series networks [37]. DeepLabV3+ is a state-of-the-art model for image segmentation tasks that has demonstrated excellent performance and accuracy in segmenting images. This architecture incorporates a powerful encoder-decoder structure, atrous convolutions, and skip connections to capture multi-scale contextual information and achieve precise segmentation results. By leveraging DeepLabV3+, we aimed to enhance the accuracy and effectiveness of our image segmentation tasks and achieve high-quality segmentation outputs, thus using a different architecture named SegX-Net. The network details are illustrated in Fig 5. The input images used in the present study are high-resolution satellite photographs that depict real-world circumstances, including prominent aircraft contrails. Segmentation aims to accurately detect and analyze these contrails in the photos, enabling a thorough investigation of their distribution and contributing to a full comprehension of their environmental influence, particularly in climate change.

### 3.1 Dataset description

**Fig 3:**
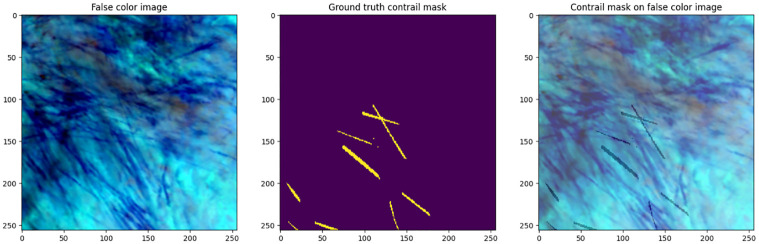
Sample input images from the dataset are showcased, providing an overview of the data variety and content.

In this study, we used a dataset obtained from Kaggle [38]. From there, we took 18000 images, which were divided into two subsets: a training set and a validation set. These images were sourced from the GOES-16 Advanced Baseline Imager (ABI) [39], and access to the original data was facilitated through Google Cloud Storage. The technical specifications of the ABI sensor, including resolution and spectral bands, are explicitly detailed to offer readers comprehensive insights into the data source. To adapt the full-disk images, bilinear resampling was applied, resulting in localized scene images. The training set comprised 14,400 images, accounting for 80 percent of the entire dataset, while the validation set contained 3,600 images, making up the remaining 20 percent. This division was crucial to ensure the effectiveness and generalizability of our model’s performance. The training set played a vital role in training our model, enabling it to learn and extract meaningful features from a diverse range of images. We also tested our model on 1000 new images that were not used during training and validation. This helps ensure the reliability and generalization capability of our model. In the dataset section, we present a diverse set of input images that play a pivotal role in our research on contrail detection, is shown in Fig 3. These images encompass various elements, including false color representations, ground truth contrail masks, and overlays of contrail masks on false color images. The false color images provide unique visual insights, showcasing essential spectral information for our analysis. Meanwhile, the ground truth contrail masks offer precise outlines of contrail regions, serving as valuable reference data for model evaluation and training. We comprehensively understand contrail distribution and spatial relationships within the original scenes by superimposing contrail masks on false color images. By utilizing such a substantial portion of the dataset for training, we optimized the model’s parameters and refined its segmentation predictions, ultimately enhancing its accuracy and performance. On the other hand, the valid set served as an independent and unseen dataset, used explicitly to evaluate our trained model’s generalization capabilities.

**Fig 4:**
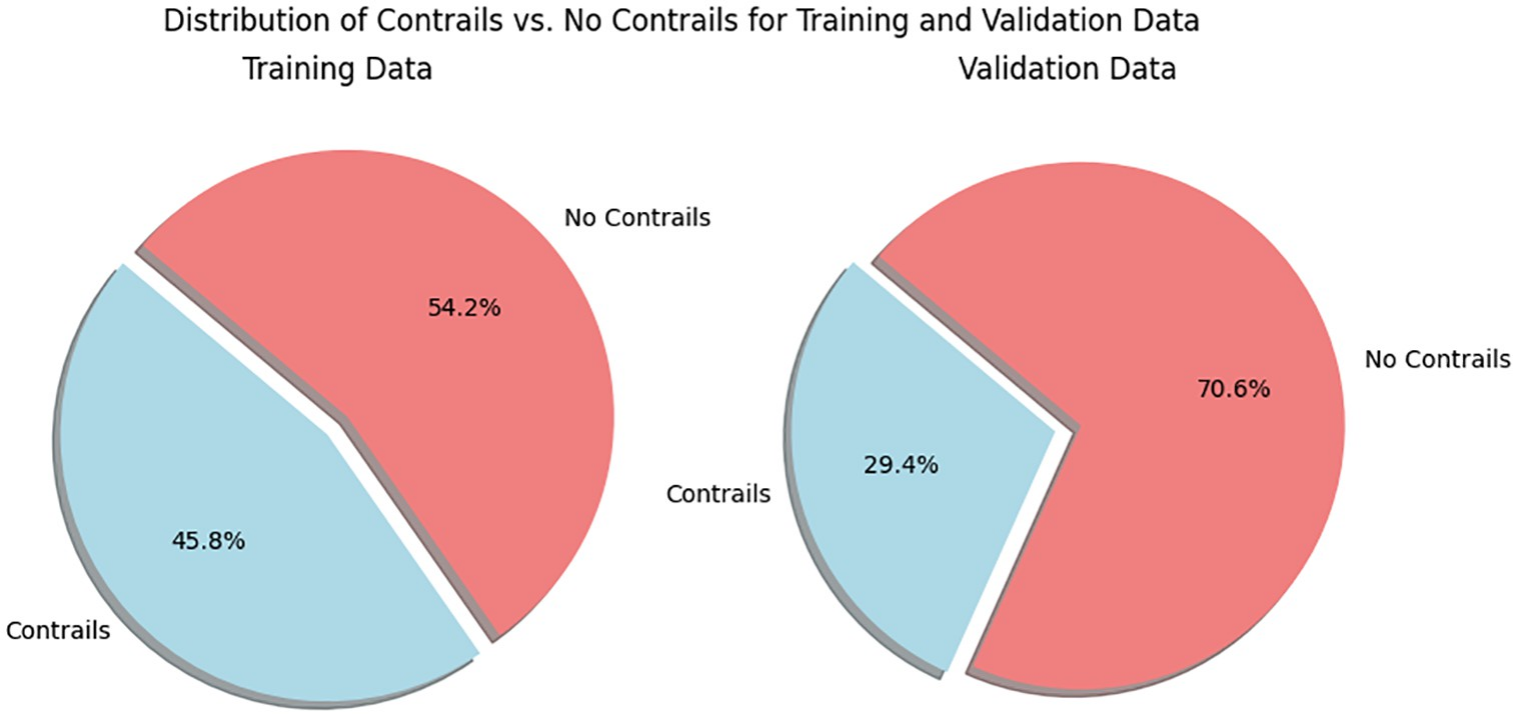
Illustration of the contrail and non-contrail distribution in the training and valid dataset split.

By assessing the model’s performance on this valid set, we obtained an unbiased estimation of its effectiveness in segmenting new, previously unseen images. The distribution of images in our training dataset shown in Fig 4 holds significant insights into the prevalence of contrail and non-contrail images. The first pie chart reveals a nuanced balance, with approximately 45.8% of images portraying contrails and 54.2% depicting scenes without contrails. This equilibrium underscores the diverse nature of our dataset, ensuring a comprehensive representation of real-world scenarios. Moreover, the second pie chart further illuminates this distribution, highlighting 29.4% percent contrail images and 70.6% percent non-contrail images. These proportions emphasize the prominence of non-contrail instances and reinforce the complexity of our task, as the model must discern and accurately segment contrails within a predominantly non-contrail context.

### 3.2 Data pre-processing

The image data underwent several crucial preprocessing steps in the initial training and validation preparation stages. Firstly, the images were converted into an array format with dimensions of 256x256, taking into account the presence of 3 color channels. The dataset was partitioned to ensure an unbiased evaluation, with 80% of the data being used for training and the remaining 20% for validation. One of the most essential steps in the preprocessing pipeline was image normalization, which aimed to establish consistency in pixel values across the dataset. The standard methods used included scaling the pixel values to a specific range, such as dividing them by the maximum value or applying a zero-mean normalization technique by subtracting the mean and dividing by the standard deviation. Implementing these preprocessing steps significantly improved the model’s resilience and adaptability, allowing it to handle various inputs and perform effectively on unseen data. It was crucial to consistently apply the same preprocessing techniques to both the training and validation sets to ensure fairness in evaluation and accurate performance assessment.

### 3.3 Proposed architecture

**Fig 5:**
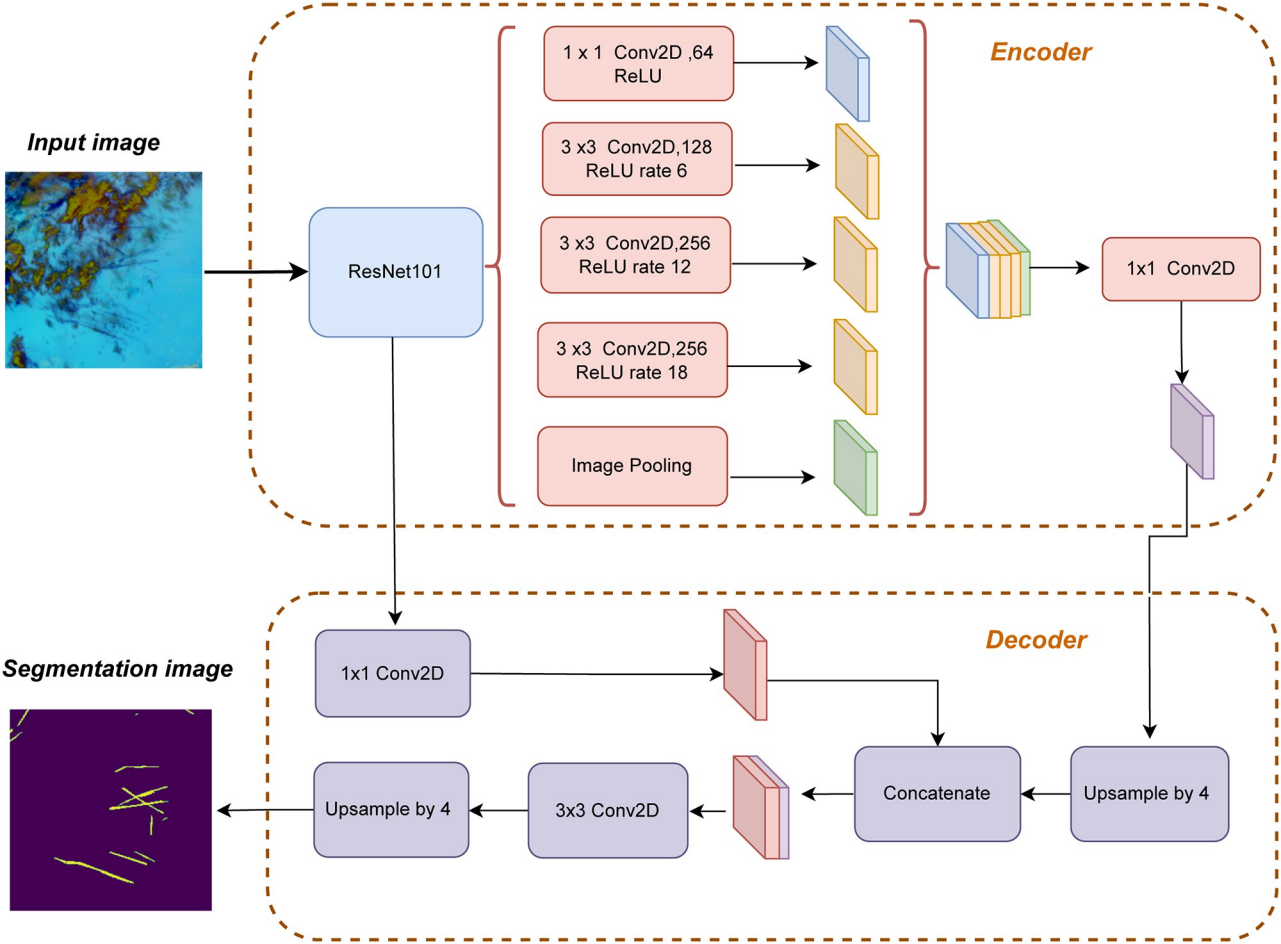
The diagram presents the architectural structure of SegX-Net, the novel model introduced in our research, delineating its key components and their interconnections.

In this work, we proposed an enhanced architecture by integrating ResNet-101 as the backbone network into the DeepLabV3+ model [31]. The decision to incorporate ResNet-101 into our model is crucial for enhancing segmentation performance in our particular environment. The deep architecture and skip connections of ResNet-101 are crucial for effectively analyzing the delicate features in satellite photos and understanding the complicated patterns of contrails. Our model’s properties enable it to accurately record and transmit detailed information, which is particularly important for identifying and describing the unique and often subtle aspects of aircraft contrails in the images. The architecture’s profound and sophisticated feature extraction processes make it a prudent choice specifically designed to meet the requirements of our contrail detection assignment, thereby improving the model’s ability to segment objects accurately. Our proposed architecture is shown in Fig 5. Our research’s encoder component of DeepLabV3+ utilizes ResNet-101 as the backbone network. By incorporating ResNet-101, our model can leverage its capabilities to extract high-level abstract information from the input image [40]. The input image is passed through convolutional blocks in ResNet-101, which perform successive convolutions and pooling operations to extract hierarchical features at different levels of abstraction. The convolutional layers capture local patterns and spatial information, while the pooling layers downsample the feature maps while retaining important features. The decoding part of DeepLabV3+ focuses on refining and enhancing the features obtained from the encoder. The shallow features from the encoder are merged with the upsampled deep features, and convolutional operations are applied to refine the feature details. This refinement process aims to improve the segmentation predictions by enhancing the feature representation. Finally, the refined features’ resolution is restored through bilinear upsampling, resulting in the final segmentation map. Fig 5 visually represents the intricate structure of the network model described in this article. The figure highlights distinct elements, with the blue and yellow sections signifying 1x1 convolutions and 3x3 convolutions, respectively. Additionally, the figure demonstrates the utilization of maximum pooling and up-sampling techniques. During the encoding phase, the network performs through a series of operations, including 1x1 convolutions, which has channels of 64, three sets of 3x3 convolutions, which have 128 channels rate 6, 256 channels rate 12, 256 channels rate 18 respectively, and also maximum pooling. These operations collectively enable the network to extract and capture meaningful features from the input data. In the decoding phase, a single set of 1x1 convolutions is employed to restore the original size of the feature map. Following this, a combination of up-sampling and 3x3 convolutions is employed to generate the final prediction image, accomplishing the image segmentation task. The figure includes annotations that specify the layer names, output feature map sizes, and the corresponding operations involved, such as Conv for convolution, image pooling for maximum pooling, and upsample for up-sampling.

### 3.4 Residual module block

The Deep Residual Network, proposed by [41], introduced the concept of residual learning. The residual refers to the discrepancy between the observed and estimated values. Assuming the input to the network is denoted as x and the expected mapping as M(x), the network mapping can be reformulated as the residual, represented by F(x), as shown in Eq (1).

$$\begin{array}{c} M(x)=F\left(x\right)+x \end{array}$$ (1)

Here, x represents the characteristic mapping of the upper layer network, F(x) denotes the residual of the current layer and M(x) represents the observed value at that layer, forming the relationship depicted in Eq (2).

$$\begin{array}{c} x_{N+1}=x_{N}+F\left(x_{N}\right) \end{array}$$ (2)

$$\begin{array}{c} x_{N}=x_{i}+\sum_{i=1}^{N-1}F\left(x_{i}\right) \end{array}$$ (3)

Although both M(x) and F(x) + x yield the same effect, optimizing F(x) is simpler compared to optimizing M(x). By considering the relationships between different layers, expressed in Eqs (3) and (4), the residual F(*x*_*N*_) is added to the previous layer’s output *x*_*N*_ to obtain the output of the current layer, *x*_*N*_ + 1.

$$\begin{array}{c} \frac{\partial Loss}{\partial Ip}=\frac{\partial Loss}{\partial Iq}+\frac{\partial Loss}{\partial Iq}*\frac{\partial }{\partial Ip}\sum_{i=1}^{N-1}F-\left(x_{i}\right) \end{array}$$ (4)

To ensure optimal network performance, it is crucial to strike a balance in terms of network depth. While a certain depth may lead to the best model performance and lowest loss, further increasing the network depth could potentially result in network degradation. To address this, the concept of the residual network is introduced, enabling the residual F(x) to approach zero and maintaining the network in an optimal state.

### 3.5 ResNet-101 encoder

**Fig 6:**
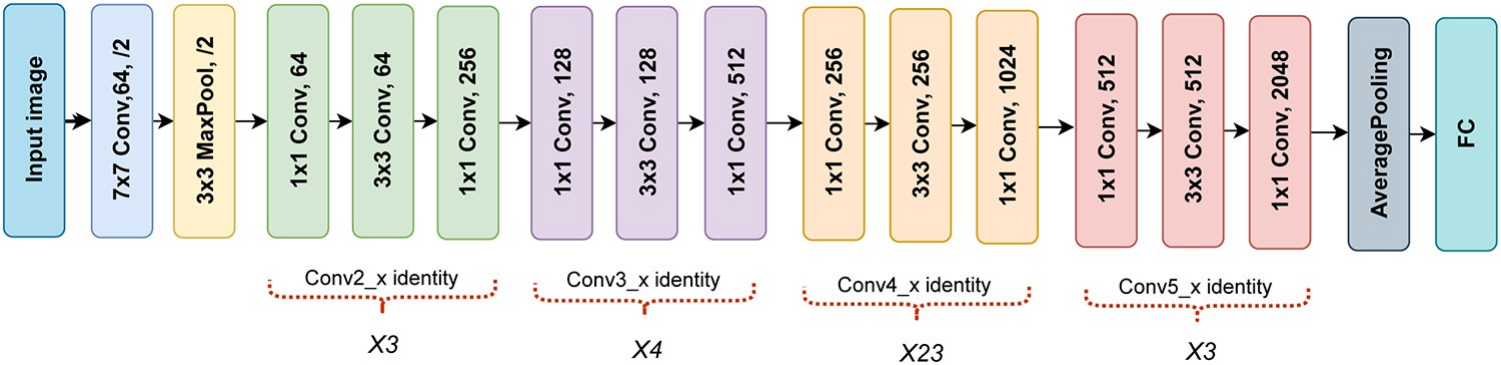
This illustration provides a visual representation of the ResNet-101 architecture, shedding light on its intricate design and layer connectivity.

In the SegX-Net architecture, ResNet-101 serves as an essential encoder, contributing to the network’s ability to capture intricate features from input images effectively shown in Fig 6. ResNet-101, introduced by [42], is a deep convolutional neural network that introduces a novel approach to addressing the vanishing gradient problem in deep neural networks. ResNet-101, a variant comprising 101 layers, employs residual blocks that facilitate extracting meaningful features. Notably, in the SegX-Net framework, ResNet-101 is not a feature extractor but an integral component for enhancing the network’s segmentation capability. ResNet-101’s architecture is characterized by its depth and structure, involving stacked residual blocks. Each residual block incorporates convolutional layers and shortcut connections bypassing certain layers. This design allows the network to adjust input features directly and learn more abstract features through subsequent layers. The architecture also integrates bottleneck structures to optimize computational efficiency by reducing convolutional layer complexities. These bottlenecks employ 1x1, 3x3, and 1x1 convolutional layers to selectively decrease input and output channels. Additionally, ResNet-101 includes global average pooling and fully connected layers at the end, culminating in a final classification or segmentation output. Although not used for feature extraction in SegX-Net, ResNet-101’s depth and shortcut connections contribute to its proficiency as an encoder in the network’s image segmentation process.

### 3.6 Architectural framework and comparative backbone models

During this study, we harnessed the power of transfer learning to enhance the performance of our image segmentation model. After a thorough evaluation of VGG16, VGG19, VIT, Xception, MobileNet_V2, ResNet18, ResNet34, and ResNet101, we found that ResNet101 exhibited superior performance and accuracy, prompting its seamless integration as an encoder into our segmentation architecture. Fine-tuning the model on our dataset allowed it to adapt to our requirements while retaining essential learned features. Adopting transfer learning addressed data limitations and expedited training, leading to improved results and heightened accuracy in our image segmentation. This integration empowered our model to capture meaningful patterns effectively, making it a pivotal element in elevating our image segmentation research’s overall performance and effectiveness. This adaptation aimed to balance capturing detailed features and maintaining computational efficiency. By integrating ResNet-101 into SegX-Net, we harnessed the enhanced capabilities of ResNet-101 while preserving the efficient encoding-decoding architecture of DeepLabV3+. This integration successfully improved segmentation accuracy, as evidenced by our experimental results. In summary, the integration of ResNet-101 into SegX-Net represents a strategic enhancement to the original architecture of DeepLabV3+. This integration allowed us to leverage cutting-edge techniques, resulting in superior segmentation performance.

In this research, we benchmarked the performance of SegX-Net against several top image segmentation architectures to conduct a thorough comparison analysis. This comprised well-known models, including DeeplabV3+, Attention U-Net, Trans U-Net, Res U-Net, U-Net++, and U-Net. We sought to identify SegX-Net’s advantages relative to these well-known architectures by comparing these models across many essential performance measures, including IoU and F1 scores. By comparing SegX-Net with cutting-edge segmentation frameworks, this comparative method shows the exceptional contributions of SegX-Net and offers insightful information about how well it detects contrails.

### 3.7 Enhanced SegX-Net model

**Fig 7:**
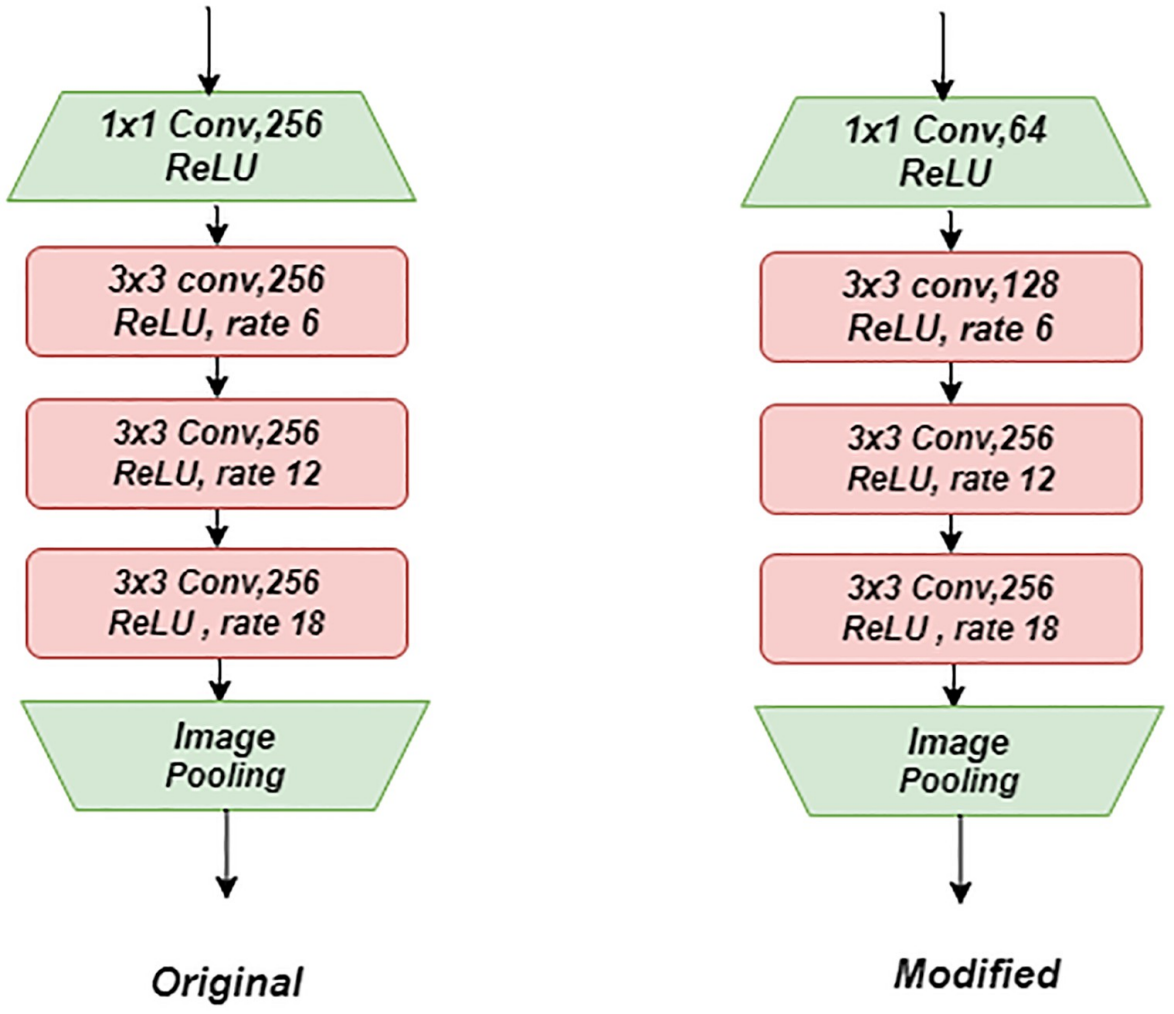
Clear visual comparison of modified and original architecture segments, showcasing enhancements in our model.

In this work, we proposed an enhanced version of the DeepLabV3+ model by modifying its encoder part with ResNet-101 shown in Fig 7. Specifically, we focused on improving the initial process by changing the first two blocks out of the original five blocks. In the first block, we replaced the 1x1 convolution followed by 256-neuron ReLU activation with a 1x1 convolution followed by 64-neuron ReLU activation—this modification aimed to reduce the dimensionality of the feature maps while preserving the relevant information. Similarly, in the second block, we replaced the 3x3 convolution followed by 256-neuron ReLU activation with a 3x3 convolution followed by 128-neuron ReLU activation, maintaining the same atrous rate of 6. This adjustment allowed for a more fine-grained feature representation at the atrous rate. We observed that these modifications in the encoder part yielded improved results compared to the original DeepLabV3+ model. The enhanced model exhibited enhanced segmentation accuracy and detail preservation, improving overall performance. Fig 7 compares the original DeepLabV3+ encoder part and our modified version. The figure highlights the changes made in the first two blocks, illustrating the altered architecture and visually representing the improved process. We conducted comprehensive evaluation experiments to quantify the performance improvement using various evaluation metrics, including IoU and F1 scores. The results demonstrate that our enhanced model outperforms the original DeepLabV3+ model, achieving higher accuracy and better segmentation results. These findings highlight the efficacy of the proposed modifications in the encoder part of DeepLabV3+ with ResNet-101 and their positive impact on the model’s overall performance. The following section presents a detailed analysis and comparison of the performance between the original DeepLabV3+ model and our enhanced version, further substantiating the superiority of the modified architecture.

## 4 Experimental results

In this research, we presented the results of our experiments evaluating the performance of the SegX-Net model for image segmentation in addressing contrail detection challenges. The dataset focused on contrails, obtained from NOAA GOES-16, was preprocessed by transforming images into 256x256 arrays with 3 color channels. Evaluation metrics included Intersection over Union (IoU), F1 score and Dice Loss as the loss function during training.

### 4.1 Evaluation metrics

To evaluate the performance of each model and effectively assess its learning capabilities, this experiment employed multiple control parameter variables for evaluation. The primary evaluation metrics included the F1 score and Intersection over Union (IoU).

#### 4.1.1 IoU

When it comes to measuring the accuracy of image segmentation, the Intersection over Union (IoU) is widely regarded as a representative evaluation metric.

$$\begin{array}{c} IoU=\frac{TP}{TP+FP+FN} \end{array}$$ (5)

IoU quantifies the overlap between the predicted values generated by the model and the true values represented by the sample labels. It provides a measure of the alignment and agreement between the predicted and ground truth segmentation masks, reflecting the accuracy and quality of the segmentation results.

#### 4.1.2 F1 score

Additionally, the F1 score provides a balanced measure of precision and recall, considering both the true positives and false positives in the segmentation predictions. Together, the F1 score and IoU offer comprehensive insights into the model’s performance and effectiveness in image segmentation tasks.

$$\begin{array}{c} F1Score=2*\frac{recall*precision}{recall+precision} \end{array}$$ (6)

#### 4.1.3 Dice loss

The Dice Loss was used in during the research as a loss function during the training of our image segmentation model. Its utilization served two primary purposes: to guide the model’s optimization process and to align the predicted segmentation masks with the ground truth masks.

$$\begin{array}{c} DiceLoss=1-\frac{2\sum_{i=1}^{k}x_{i}*y_{i}}{\sum_{i=1}^{k}x_{i}+\sum_{i=1}^{k}y_{i}} \end{array}$$ (7)

The Dice Loss was an integral part of our research, serving as a key component in optimizing the model’s performance and enhancing the accuracy and quality of our image segmentation results. Its usage helped align our model’s predictions with the ground truth, ultimately leading to improved segmentation accuracy and precise delineation of objects in the resulting segmentation masks. In the equation. Xi indicates the predicted target category and yi indicates the actual target category.

#### 4.1.4 Dice coefficient

Within the realm of image segmentation, where computers manipulate pixels and reality wields the brush, the Dice coefficient arises as a reliable measure of precision. It functions as a measurement tool to assess the degree of overlap between a predicted area, such as a tumor or a contrail, and the actual region. The correlation between the Dice score and the fit is directly proportional, indicating a stronger alignment between prediction and reality as the Dice score increases.

$$\begin{array}{c} DiceCoefficient=\frac{2TP}{2TP+FN+FP} \end{array}$$ (8)

#### 4.1.5 Sensitivity analysis

Sensitivity in image segmentation, measures the algorithm’s ability to correctly identify positive instances, crucial in applications like medical imaging. A sensitivity value close to 1 indicates effective detection of relevant regions, while lower values suggest potential misses. Sensitivity analysis involves varying parameters to understand how the algorithm responds to changes, aiding optimization for specific applications.

$$\begin{array}{c} Sensitivity=\frac{TP}{TP+FN} \end{array}$$ (9)

### 4.2 Efficiency analysis and comparison

An essential aspect of evaluating the SegX-Net architecture’s effectiveness is assessing its computational efficiency in contrast to existing models. This analysis sheds light on the architectural optimizations that SegX-Net introduces. Notably, a comparative examination of parameter sizes between SegX-Net and the default DeepLabV3+ model with ResNet-101 showcases SegX-Net’s streamlined design, boasting a parameter count of 33,815,745 as opposed to the default model’s 35,278,721. This reduction in parameter count signifies SegX-Net’s potential for optimized memory usage, as evident by its memory size of 129 MB compared to DeepLabV3+ with ResNet101’s 135 MB. Furthermore, when considering time complexity, SegX-Net demonstrates its efficiency by achieving an average processing time of 694ms per iteration, iterated ten times on average, while the default DeepLabV3+ with ResNet-101 requires 901ms. This rigorous analysis underscores SegX-Net’s computational superiority, making it a promising solution for enhancing contrail detection efficiency and outperforming existing models in terms of both memory utilization and processing speed.

### 4.3 Parameter analysis

For evaluating the model, we employ key metrics such as Intersection over Union (IoU), F1-score and Dice Loss, Dice Coefficient and sensitivity analysis. The results are presented through insightful line plots, illustrating the Dice Loss, IoU and F1-score, Dice Coefficient and sensitivity analysis for both the training and valid sets over epochs. We have showcased the quantitative results obtained from the training and valid sets.

**Fig 8:**
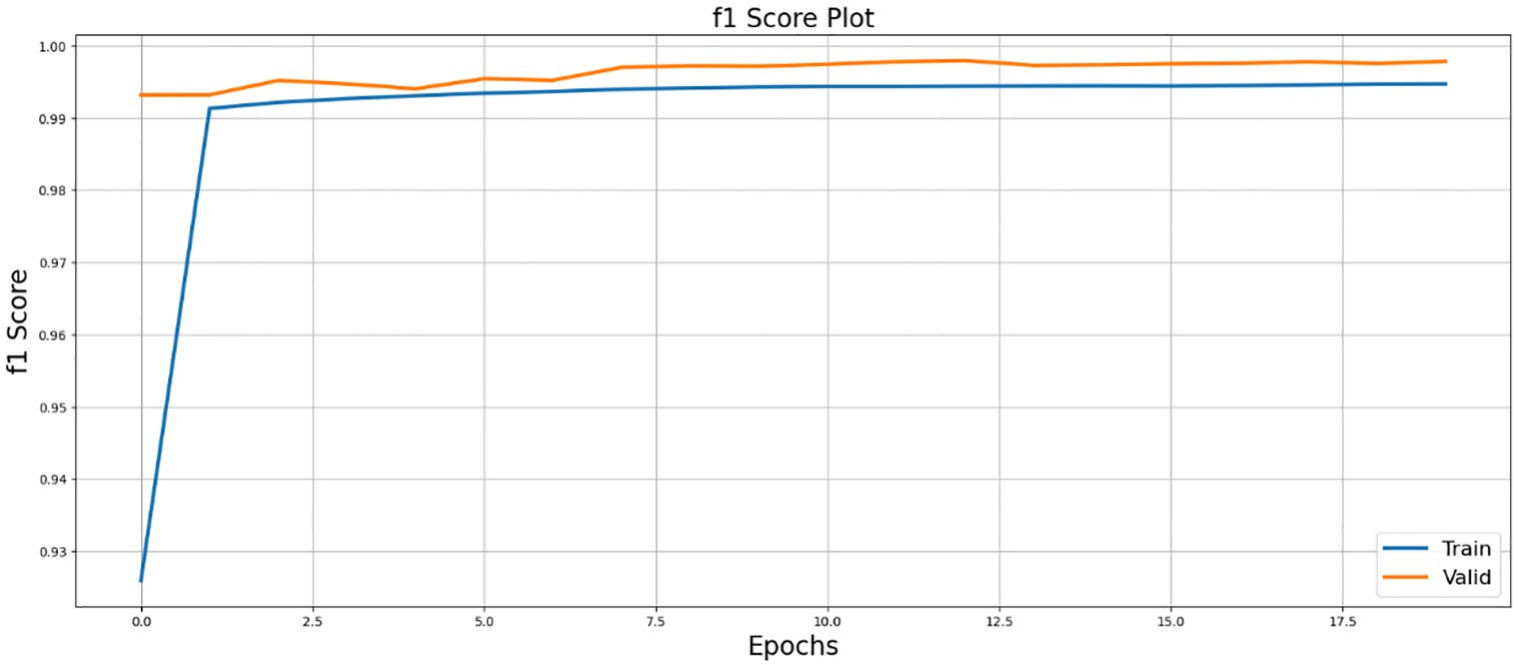
Illustration of F1-score visualization, demonstrating its utility in quantifying the performance of SegX-Net.

Fig 8 presents the F1 score plot, which balances precision and recall, reflecting the model’s segmentation performance.

**Fig 9:**
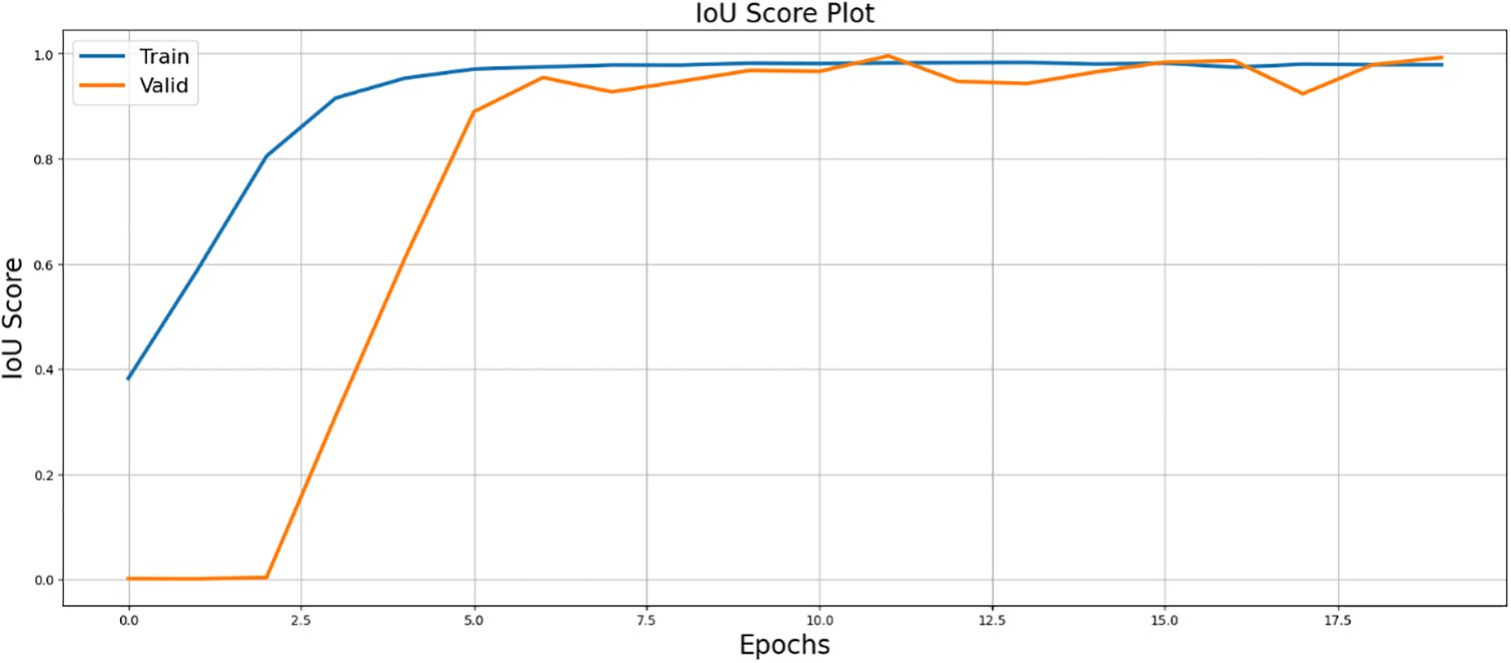
Visualization of SegX-Net performance using Intersection over Union (IOU) metric, showcasing the effectiveness of the model.

Fig 9, displays the IoU plot, highlighting the model’s accuracy in capturing object boundaries and overall segmentation quality.

**Fig 10:**
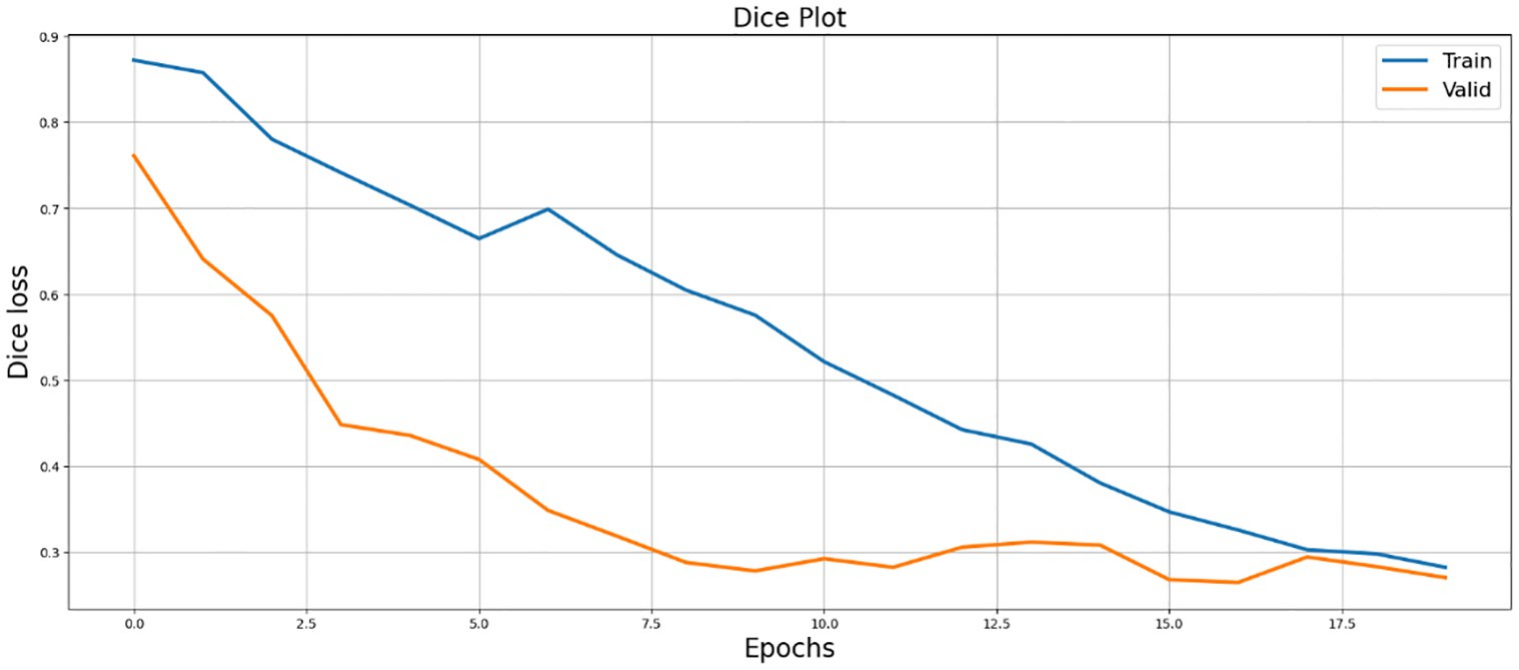
Illustrating the performance evaluation of SegX-Net through the utilization of Dice loss, providing insights into the model’s efficacy.

Fig 10, illustrates the Dice Loss plot, providing insights into the model’s optimization process and alignment with ground truth masks.

**Fig 11:**
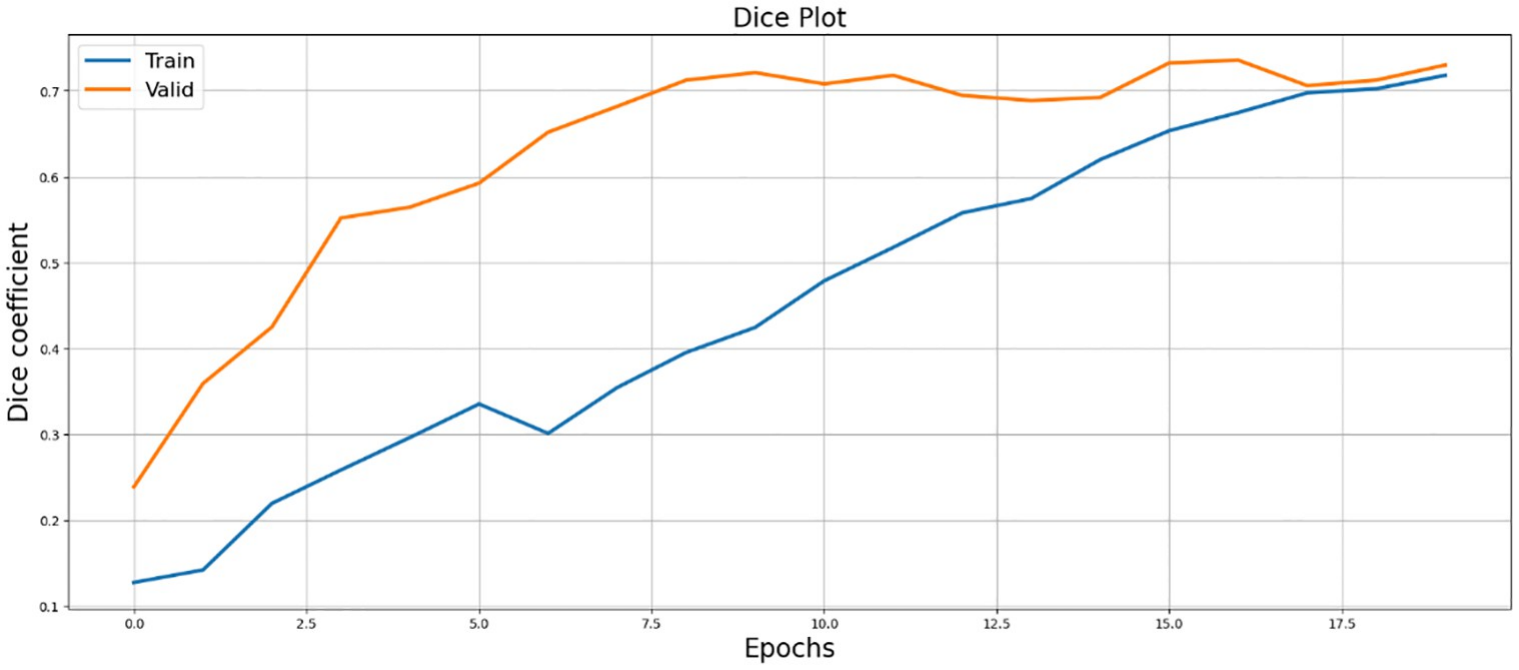
Illustrating SegX-Net’s efficacy through Dice coefficient performance evaluation.

The effectiveness of SegX-Net is shown in Fig 11 the use of dice coefficient performance assessment.

**Fig 12:**
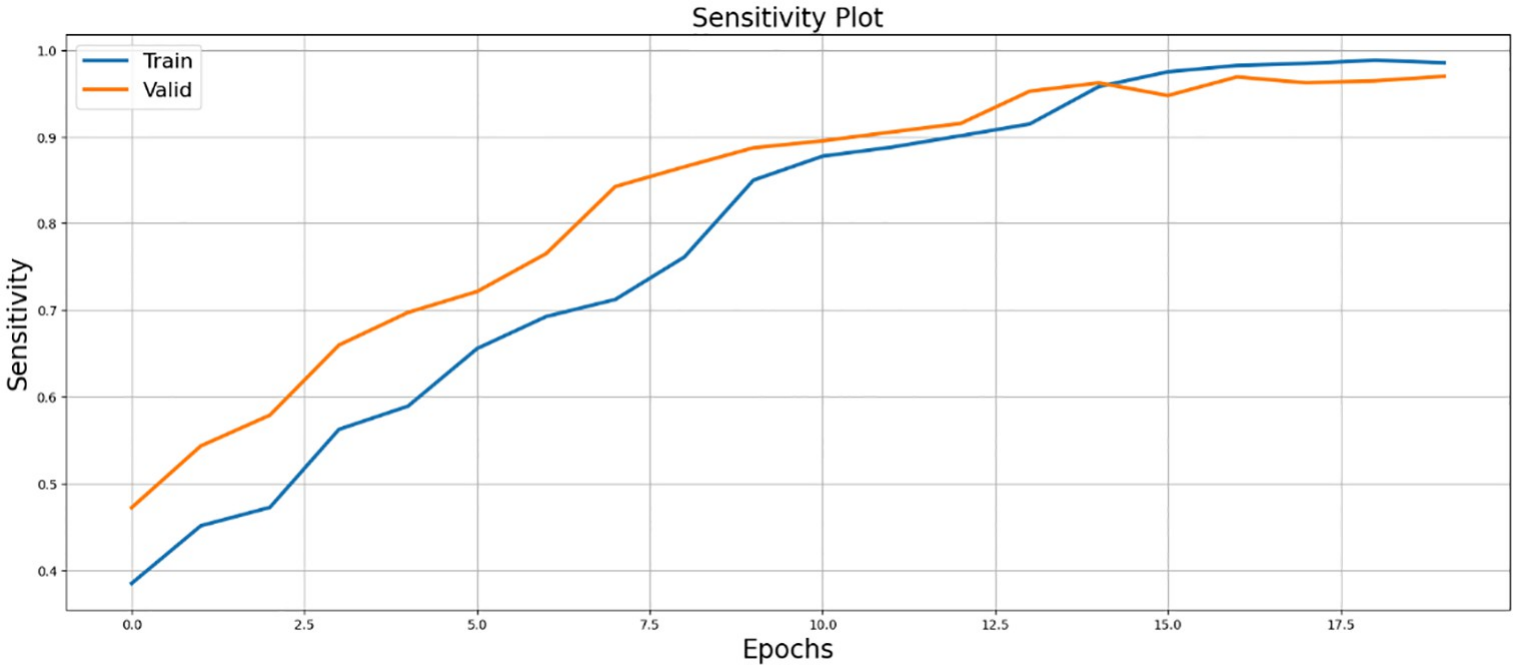
Upon sensitivity analysis, SegX-Net demonstrated exceptional true positive detection and minimal false negatives, confirming its efficacy in image segmentation.

The results of the sensitivity study shown in Fig 12 revealed that SegX-Net had an extraordinary true positive detection rate and a low number of false negatives, which confirmed its effectiveness in picture segmentation work.

### 4.4 Comparative experiment

In the results section, our experiments with the SegX-Net model unveil remarkable outcomes, showcasing an exceptional F1 score of 99.47% (Table 1) and an impressive IoU score of 98.86% and (Table 2). These scores, complemented by the Dice Loss plot (Fig 10) offering insights into the model’s optimization, the captivating IoU plot (Fig 8) demonstrating its unprecedented accuracy and the striking F1 score plot (Fig 9), collectively attest to the revolutionary capabilities of SegX-Net. Additionally, we have introduced Dice Coefficient (Fig 11) and Sensitivity Analysis (Fig 12), further solidifying the model’s exceptional segmentation performance. These extraordinary achievements establish SegX-Net as a pioneering solution, advancing contrail detection through cutting-edge artificial intelligence. The remarkable sensitivity, notably during training with 98.82%, shown in (Table 2), that the model can recognize most contrails in images. As we balance sensitivity and accuracy, we must consider environmental monitoring’s contrail detection goals. Although our SegX-Net achieves a slightly lower Dice Coefficient score on the training set compared to VGG16 (71.79% vs. 72.61%), it is crucial to take into account the larger context. Our model exhibits improved performance compared to VGG16, as shown by a Dice Coefficient score of 73.55% on the validation set. The validation set serves as a crucial indicator of generalization. This review highlights the strong and flexible nature of SegX-Net in accurately capturing the complex patterns of contrails. Although training performance is important, the validation results emphasize the model’s effectiveness in real-world situations, confirming its status as an advanced solution for contrail identification.

**Table 1:** Comparison results of model’s backbone with different architectures based on IoU scores and dice coefficient.

Model Backbone	Train (IoU)	Val (IoU)	Train (Dice)	Val (Dice)
VGG16 [28]	82.97%	72.11%	**72.61%**	56.62%
VIT [30]	97.56%	86.45%	59.50%	56.62%
VGG19 [29]	85.21%	82.92%	67.84%	51.23%
Xception [31]	88.87%	92.51%	42.72%	40.54%
ResNet 18 [32]	89.62%	99.28%	45.95%	35.83%
ResNet 34 [33]	92.47%	96.88%	60.45%	61.65%
ResNet 101 [34]	96.97%	96.13%	59.53%	54.48%
MobileNet_V2 [35]	95.72%	94.88%	68.48%	70.19%
**SegX-Net**	**98.86%**	**99.54%**	71.79%	**73.55%**

**Table 2:** Comparison results of models backbone with different architectures based on F1-scores and sensitivity.

Model Backbone	Train(F1)	Val(F1)	Train(Sensitivity)	Val(Sensitivity)
VGG16 [28]	88.85%	80.00%	85.87%	88.68%
VGG19 [29]	91.44%	86.57%	88.21%	79.39%
VIT [30]	96.56%	87.67%	76.56%	66.91%
Xception [31]	94.82%	92.98%	80.72%	82.99%
ResNet18 [32]	96.25%	94.93%	87.95%	85.27%
ResNet34 [33]	97.58%	96.29%	96.59%	91.82%
MobileNet_V2 [35]	97.71%	97.92%	95.68%	93.91%
ResNet101 [34]	97.45%	96.21%	91.70%	92.58%
**SegX-Net**	**99.47%**	**99.77%**	**98.82%**	**96.97%**

Here, we have shown a comprehensive analysis of the performance metrics for each model on both Intersection over Union (IoU), Dice-Coefficient in Table 1 and F1-score, Sensitivity Score in Table 2.

Table 1 provides an in-depth comparison of the IoU scores obtained by the different models on both the training and valid sets. The results clearly illustrate the exceptional segmentation accuracy achieved by SegX-Net, with an outstanding IoU score of 98.86% on the training set and an impressive 99.54% on the valid set. This demonstrates the model’s ability to accurately capture object boundaries and produce high-quality segmentations, outperforming all other models, including DeepLabV3+ with VGG16, VGG19, VIT, Xception, MobileNet_V2, ResNet18, ResNet34 and ResNet101.

In Table 2, we delve into the F1-scores of each model on the training and valid sets, shedding light on their segmentation performance in finer detail. The results reveal the exceptional capabilities of SegX-Net, achieving a remarkable F1-score of 99.47% on the training set and an extraordinary 99.77% on the valid set. These scores surpass the performance of DeepLabV3+ with VGG16, VGG19, VIT, Xception, MobileNet_V2, ResNet18, ResNet34 and ResNet101 by a substantial margin. Such exceptional F1-scores indicate the model’s precision and recall capabilities, affirming its ability to achieve superior segmentation results.

Our SegX-Net model significantly outperforms these models in terms of Intersection over Union (IoU) scores when compared to a group of cutting-edge competitors shown in Table 3, including U-Net, U-Net++, Attention U-Net, Trans U-Net, Res U-Net, Uc Trans U-Net, and DeeplabV3+. The results demonstrate our proposed SegX-Net’s better accuracy and efficiency in separating contrail clouds. In climate research, aviation studies, and environmental monitoring, where the precision of contrail cloud identification is crucial, SegX-Net demonstrates a greater capacity to properly identify contrail areas with a higher IoU score. As a result, it is a useful tool in these fields.

**Table 3:** Comparison results of models with different architectures based on IoU scores and dice coefficient.

Model	Train (IoU)	Val (IoU)	Train (Dice)	Val (Dice)
U-Net [43]	96.48%	90.25%	49.96%	39.12%
U-Net++ [44]	95.95%	92.45%	41.84%	35.83%
Attention U-Net [45]	96.72%	88.51%	47.98%	44.88%
Trans U-Net [31]	96.62%	91.38%	57.77%	51.92%
Res U-Net [46]	96.88%	95.93%	**75.34%**	59.11%
Uc Trans U-Net [47]	97.09%	95.74%	60.63%	55.89%
DeeplabV3+ [48]	97.48%	97.68%	70.12%	56.94%
**SegX-Net**	**98.86%**	**99.54%**	71.79%	**73.55%**

In parallel evaluations of the same models, we focus on F1 scores, a statistic that balances accuracy and recall in contrail cloud segmentation. SegX-Net is the clear winner once again as shown in Table 4, outperforming U-Net, U-Net++, Attention U-Net, Trans U-Net, Res U-Net, Uc Trans U-Net, and DeeplabV3+. SegX-Net’s superior F1 scores highlight its remarkable accuracy and recall skills in recognizing contrail clouds. These findings highlight the resilience of our proposed model, establishing it as a top option for accurate contrail cloud segmentation tasks, while its strong F1 scores indicate its use in aviation, and environmental impact assessments.

**Table 4:** Comparison results of models with different architectures based on F1-scores and sensitivity.

Model	Train(F1)	Val(F1)	Train(Sensitivity)	Val(Sensitivity)
U-Net [43]	88.62%	90.95%	97.67%	96.02%
U-Net++ [44]	92.92%	93.52%	90.22%	87.82%
Attention U-Net [45]	89.75%	92.59%	91.55%	95.47%
Trans U-Net [31]	95.88%	92.48%	82.79%	76.22%
Res U-Net [46]	96.75%	86.65%	96.89%	94.65%
UC Trans U-Net [47]	95.56%	90.11%	87.09%	89.18%
DeeplabV3+ [48]	97.58%	94.93%	94.22%	92.41%
**SegX-Net**	**99.47%**	**99.77%**	**98.82%**	**96.97%**

**Fig 13:**
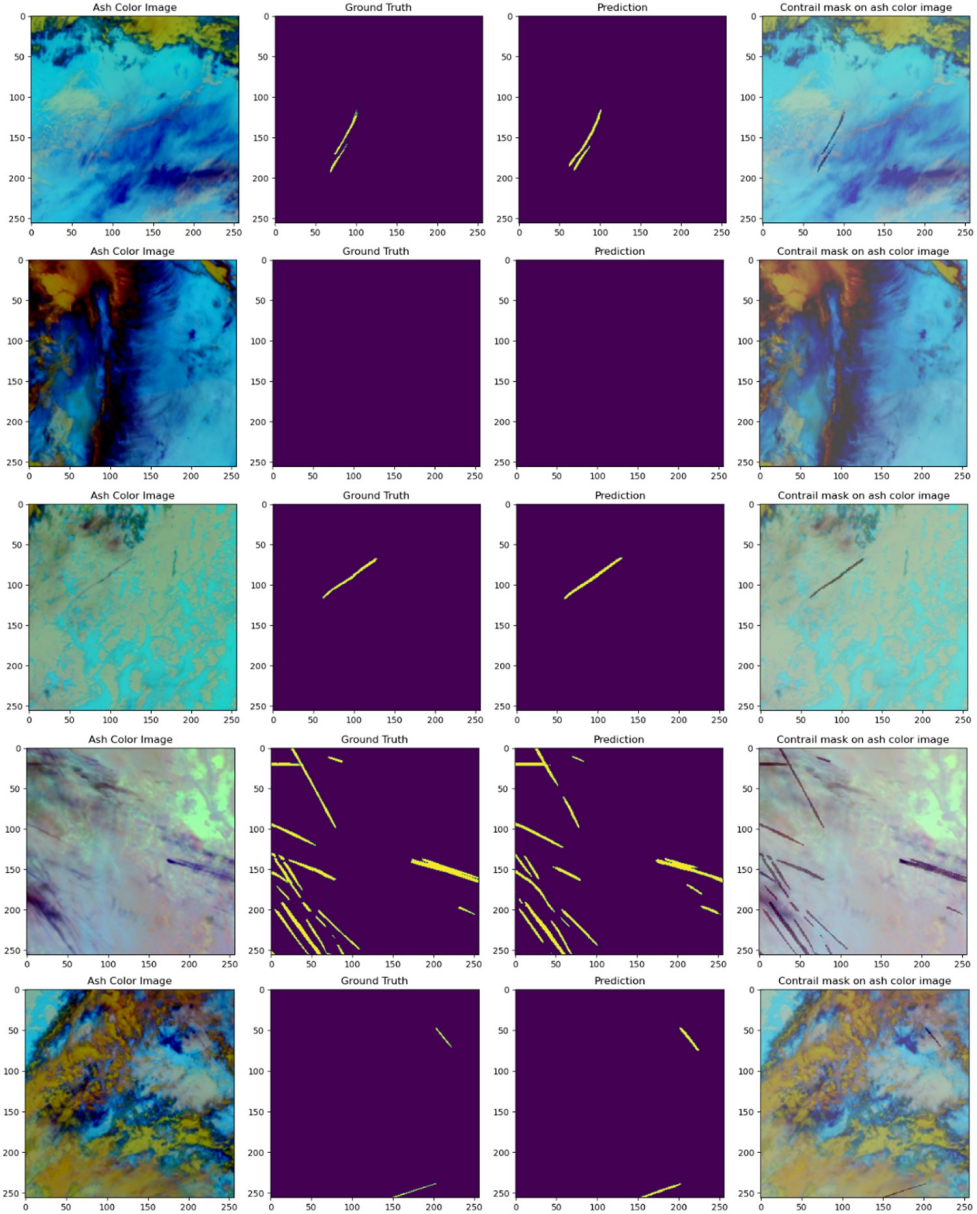
Visualization of prediction results on our dataset showcasing prediction images generated by our model on the dataset.

These comparative results are a testament to the revolutionary advancements achieved by SegX-Net in image segmentation for contrail detection. Our model has achieved outstanding performance with a remarkable IoU score of 97.90%, an F1 score of 99.51%, a Dice coefficient of 71.79%, and an impressive sensitivity of 98.41% from the testing set. These results are a testament to the reliability and accuracy of our model. The model’s extraordinary performance and accuracy set new standards in artificial intelligence and contribute significantly to addressing the challenges of climate change. The combination of state-of-the-art techniques and innovative methodologies within SegX-Net positions it as a leading solution for climate researchers and environmentalists seeking accurate and reliable image segmentation results. We present compelling prediction images from our test set, as illustrated in Fig 13. These images include Ash color, Ground truth, Prediction, and Contrail mask on Ash color. The false-color representations vividly capture essential spectral information, enhancing our analysis. The ground truth contrail masks offer precise outlines for rigorous model evaluation, serving as crucial reference data. These prediction images vividly demonstrate our contrail detection methods’ exceptional performance and accuracy, emphasizing our dedication to addressing challenges through innovative and unconventional AI approaches.

The false colour images offer vivid visual representations, capturing essential spectral information relevant to our analysis. The ground truth contrail masks serve as crucial reference data, providing precise outlines of contrail regions for rigorous model evaluation and validation. Furthermore, by superimposing the contrail masks on false colour images, we gain valuable insights into the spatial distribution and correlations of contrails within their original scenes. These prediction images showcase the remarkable performance and accuracy of our contrail detection methods, underscoring our commitment to addressing challenges through innovative and non-typical AI approaches.

## 5 Discussion

Results of our experiment emphasize the influence of enhanced segmentation methods in identifying contrails. The SegX-Net model, carefully developed and evaluated, exhibited exceptional performance metrics, attaining an IoU score of 98.86% and 99.54% and an F1-Score of 99.47% and 99.77% on the training and validation sets, respectively. The Dice Coefficient and Sensitivity scores serve to highlight the model’s reliability, in addition to the aforementioned measures. Our model has exceptional segmentation accuracy and sensitivity to complex contrail patterns, as seen by its Dice Coefficient scores of 71.79% on training and 73.55% on validation, along with a Sensitivity score of 98.82% on training.

When comparing SegX-Net to well-known models like U-Net, U-Net++, Attention U-Net, Trans U-Net, Res U-Net, Uc Trans U-Net, and DeeplabV3+, SegX-Net demonstrates superiority. Our algorithm frequently surpassed these standards, showcasing superior accuracy in contrail segmentation. This comparison confirms the effectiveness of SegX-Net and offers valuable insights into the model’s competitive advantage. SegX-Net stands out among contrail identification technologies because of its exceptional ability to identify subtle characteristics in contrails and its outstanding accuracy metrics.

In the future, it would be beneficial to investigate the scalability and generalizability of SegX-Net. Expanding the dataset to include a broader range of geographical regions and weather conditions could strengthen the model’s resilience. Moreover, the incorporation of sophisticated metrics and the investigation of adversarial training methods, such as Generative Adversarial Networks (GANs), show potential for enhancing the precision of the model. These considerations broaden the range of situations where SegX-Net can be used and contribute to the ongoing discussion on advanced methods for detecting contrails, which has significant consequences for environmental preservation.

## 6 Conclusions

In conclusion, this research paper has explored the integration of image segmentation techniques, specifically the SegX-Net architecture, to address climate change challenges related to aircraft contrails. By leveraging the DeepLabV3+ model as the baseline and integrating ResNet101 as the backbone network, we have demonstrated the effectiveness of the enhanced model in accurately identifying and segmenting contrails. The proposed modifications in the encoder part, along with transfer learning and data preprocessing techniques, have resulted in improved segmentation accuracy and detail preservation. The evaluation metrics, including IoU and F1 score, validate the superior performance of the SegX-Net architecture compared to the original DeepLabV3+ model. Overall, this research highlights the potential of image segmentation techniques in understanding and mitigating the environmental impact of aircraft contrails, contributing to the broader goal of combating climate change. Future work can focus on further refining the SegX-Net architecture and exploring additional applications of image segmentation in addressing climate change challenges.
